# Supplementary material for: New Microbe Killers: Self-Assembled Silver(I) Coordination Polymers Driven by a Cagelike Aminophosphine
Source: Materials (Basel). 2019 Oct 15;12(20):3353. doi: 10.3390/ma12203353 (PMC6829320; doi:10.3390/ma12203353)
Supplement: Supplementary file 1 [file materials-12-03353-s001.pdf]

materials

Supplementary Materials

New Microbe Killers: Self-Assembled Silver(I) Coordination Polymers Driven by a Cagelike Aminophosphine

Sabina W. Jaros [1], Matti Haukka [2], Magdalena Florek [3], M. Fátima C. Guedes da Silva [4,*], Armando J. L. Pombeiro [4], Alexander M. Kirillov [4,5,*] and Piotr Smoleński [1,*]

1 Faculty of Chemistry, University of Wrocław, ul. F. Joliot-Curie 14, 50-383 Wrocław, Poland; sabina.jaros@chem.uni.wroc.pl
2 Department of Chemistry, University of Jyväskulä, FIN-40014, Jyväskulä, Finland; matti.o.haukka@jyu.fi
3 Department of Pathology, Wrocław University of Environmental and Life Sciences, ul. Norwida 31, 50-375 Wrocław, Poland, magdalena.florek@upwr.edu.pl
4 Centro de Química Estrutural, Instituto Superior Técnico, Universidade de Lisboa, Av. Rovisco Pais, 1049–001 Lisbon, Portugal, pombeiro@tecnico.ulisboa.pt
5 Research Institute of Chemistry, Peoples' Friendship University of Russia (RUDN University), 6 Miklukho-Maklaya st., 117198 Moscow, Russia
* Correspondence: fatima.guedes@tecnico.ulisboa.pt (M.F.C.G.S.); kirillov@tecnico.ulisboa.pt (A.M.K.); piotr.smolenski@chem.uni.wroc.pl (P.S.); Tel.: +48 713757225 (P.S.)

Supporting Information (SI)

Supporting Information contains additional PXRD patterns (Figures S1–S2).

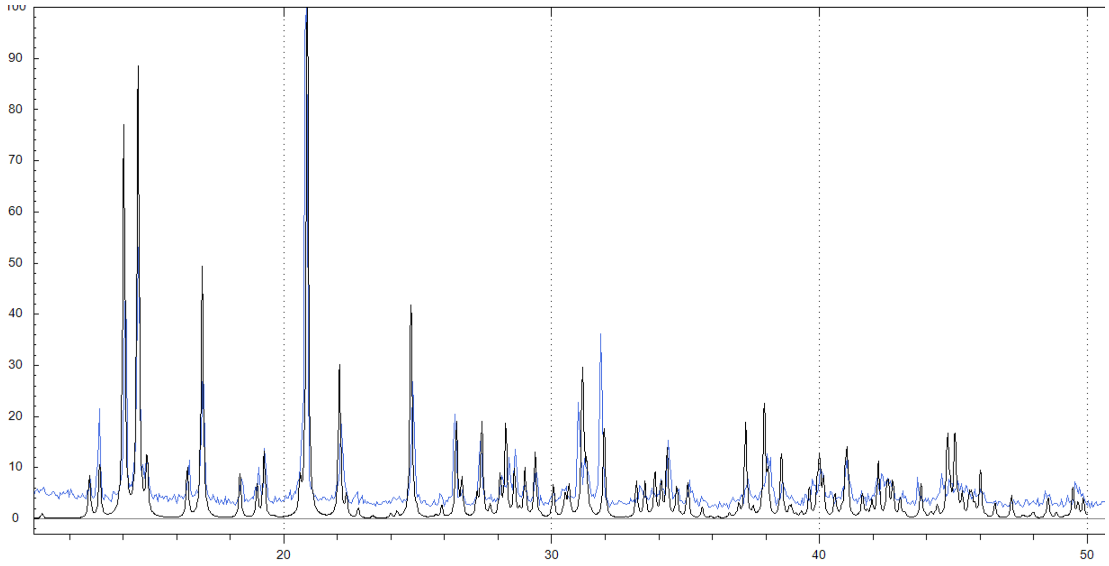

Figure S1. Comparison of PXRD pattern (black) calculated from the single crystal structure with bulk product (blue) of compound **1.**

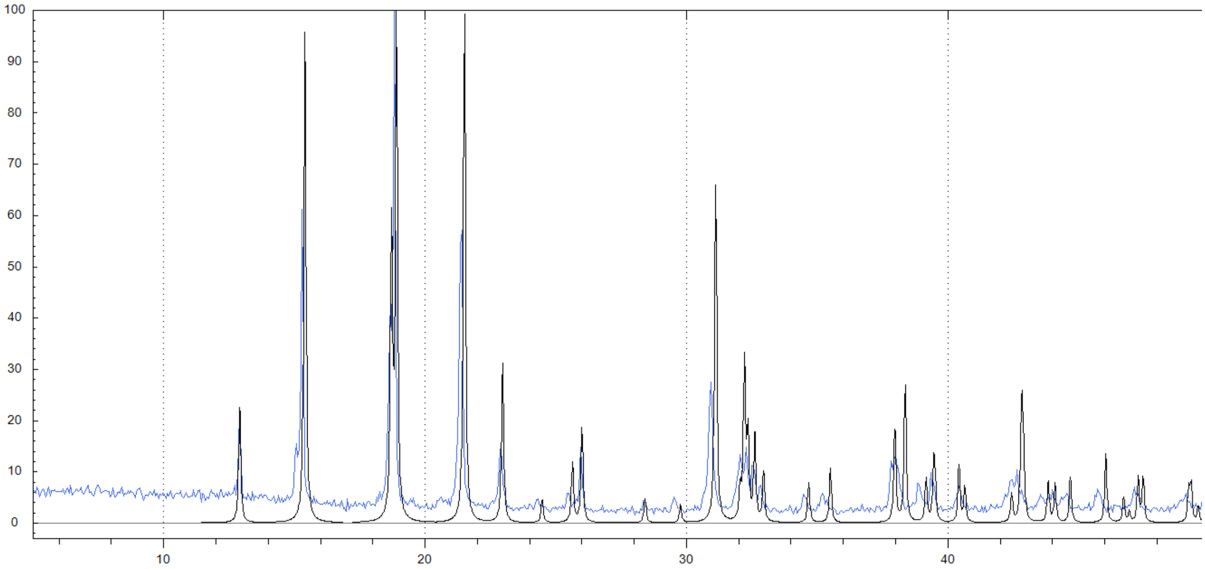

Figure S2. Comparison of PXRD pattern (black) calculated from the single crystal structure with bulk product (blue) of compound **2.**
